# SOX2, PIWI proteins, and MALAT1 – plasma-based emerging biomarkers for cancer detection and monitoring

**DOI:** 10.1371/journal.pone.0328557

**Published:** 2025-07-17

**Authors:** Ekaterina Kldiashvili, Ivane Abiatari, Elene Kekelia, Saba Iordanishvili, Tornike Metreveli, Eter Dumbadze

**Affiliations:** 1 Petre Shotadze Tbilisi Medical Academy, Ketevan Tsamebuli avenue 51/2, Tbilisi, Georgia; 2 Institute of Medical and Public Health Research, Ilia State University, Ilia Chavchavadze Avenue 32, Tbilisi, Georgia; UT Health San Antonio: The University of Texas Health Science Center at San Antonio, UNITED STATES OF AMERICA

## Abstract

**Background:**

SOX2, PIWI proteins, and MALAT1 are molecular regulators implicated in cancer progression, proliferation, and epithelial-mesenchmal transition (EMT). This study evaluated their expression in plasma samples from patients with colorectal, breast, and prostate cancers, and assessed their correlations with standard immunohistochemical (IHC) markers.

**Methods:**

A total 300 participants were enrolled: 150 patients with histologically confirmed cancers (50 colorectal cancer, 50 breast cancer, and 50 prostate cancer cases) and 150 age- and sex-matched healthy controls. Plasma RNA and protein levels of SOX2, PIWIL1, PIWIL2, and MALAT1 were measured via quantitative real-time polymerase chain reaction (qRT-PCR) and enzyme-linked immunosorbent assay (ELISA), respectively. IHC scores (Ki-67, p53, E-cadherin, vimentin, estrogen receptor/progesterone receptor, human epidermal growth factor receptor 2, androgen receptor) were retrieved from clinical records. Receiver-operating characteristic curve (ROC) analysis, multivariable logistic regression (adjusting for age and sex), and Pearson’s correlation coefficients were used to evaluate biomarker diagnostic performance and tumor marker associations.

**Results:**

SOX2, PIWI proteins, and MALAT1 were significantly elevated in cancer patients versus controls (*p* < 0.001), with qRT-PCR and ELISA results strongly correlated. All three biomarkers showed strong positive correlations with Ki-67 (r = 0.65–0.72, *p* < 0.001), and MALAT1 was associated with EMT marker changes (↓E-cadherin, ↑ vimentin; *p* < 0.001). Adjusted ROC analysis yielded area under the curve (AUC) values of 0.82–0.89 for individual biomarkers, with sensitivity ranging from 72−84% and specificity from 75−87%. SOX2 levels showed significant correlations with Ki-67 and p53 IHC positivity in colorectal and breast cancer tissues (p < 0.01), although the functional significance of p53 staining remains inconclusive*.*

**Conclusion:**

The differential expression of SOX2, PIWI proteins, and MALAT1 between cancer patients and healthy controls supports their potential utility as plasma-based biomarkers for distinguishing cancer cases from non-cancer cases. These findings support their potential utility as non-invasive biomarkers for distinguishing cancer cases from healthy individuals.

## Introduction

Cancer remains a major global health challenge, with an estimated 19.2 million new cases in 2020 [1] projected to rise to 29 million by 2040 [2]. Georgia mirrors these trends, reporting 9,435 new cancer cases and 8,024 deaths reported in 2020, with 68% of cases in individuals aged 30–70 [3]. Despite progress in imaging and histopathology, the need persists for sensitive, non-invasive biomarkers to improve early detection, risk stratification, and monitoring [4–9]. SOX2 [10,11], PIWI proteins [12–14], and MALAT1 [15–17] have been identified as potential regulators of tumor progression, offering promise for their application as plasma-based biomarkers. **SOX2** (Sex-determining region Y-box 2) is a transcription factor encoded by the gene located on chromosome 3p26.3-q27. It is essential for maintaining the stemness and pluripotency of embryonic stem cells (ESCs) during development. Since its identification as a key regulator of stem cell properties [18,19], SOX2 has been shown to be overexpressed in at least 25 types of human cancer [20], driving tumorigenesis through increased cell proliferation, migration, invasion, and metastasis [10]. SOX2 was selected over OCT4 due to its higher detectability in plasma, independent oncogenic role in epithelial tumors, and documented expression in colorectal, breast, and prostate cancers, making it a more relevant candidate for plasma-based screening in these malignancies. Moreover, high SOX2 expression is often linked to drug resistance and poor patient prognosis, establishing it as a compelling biomarker for cancer diagnosis and treatment monitoring [21]. Nonetheless, the regulatory mechanisms that control SOX2 activity in cancer remain incompletely understood. **PIWI proteins**, a subfamily of the Argonaute protein family, are crucial for maintaining genomic stability through their interactions with PIWI-interacting RNAs (piRNAs) [22]. Normally restricted to germline tissues, PIWI proteins have been aberrantly expressed in various cancers, where they contribute to tumor development by promoting cell proliferation, inhibiting apoptosis, and enhancing metastatic potential [23]. These proteins also influence epigenetic regulation and support the self-renewal of cancer stem cells, emphasizing their significance as potential markers of cancer aggressiveness and resistance to therapy [22,24]. **MALAT1** (Metastasis-Associated Lung Adenocarcinoma Transcript 1), a long non-coding RNA encoded on chromosome 11q13.1, was first identified in non-small cell lung cancer (NSCLC) due to its upregulation in highly metastatic tumors [25,26]. Subsequent studies have shown that MALAT1 is implicated in tumor initiation, progression, and resistance to chemotherapy across multiple cancer types [15]. Its highly conserved nature and multifaceted role in cancer biology have made MALAT1 a focal point for research into mechanisms of metastasis and therapeutic resistance.

Previous studies have explored tissue-level expression of these factors; however, their potential as circulating biomarkers in plasma remains underexplored. This study aims to quantify SOX2, PIWI proteins, and MALAT1 in plasma samples and evaluate their correlation with IHC-based tumor markers to assess their diagnostic potential. Additionally, we apply multivariable and ROC-based analysis to evaluate their utility across patients subgroups.

## Materials and methods

### Study design and sample collection

This cross-sectional study conducted from 1^st^ March 2024–1^st^ November 2024, included a total of 300 participants (Table 1), comprising 50 cases of colorectal cancer (25 females and 25 males), 50 cases of breast cancer, 50 cases of prostate cancer, and a control group of 150 healthy individuals (75 females and 75 males). Blood samples were collected from all participants, with the cancer cases being histologically confirmed prior (from 1^st^ March 2024–1^st^ June 2024) to the study. Ethical clearance was secured from the Bioethics International Committee of the Petre Shotadze Tbilisi Medical Academy (IRB20042024/1, Tbilisi, Georgia). All procedures adhered to the Helsinki Declaration of 1975, revised in 2013, with participants receiving comprehensive study information and providing written informed consent prior to inclusion. Study didn’t include minors. All data were fully anonymized before access by the research team, ensuring that no personally identifiable information could be linked to the participants. The process of obtaining written informed consent included providing participants with detailed information about the study’s purpose, procedure, risks, and benefits, and allowing sufficient time for questions before signing the consent form. An impartial witness was present during the consent process to confirm that participants understood the study and voluntarily agreed to participate.

**Table 1:** Sample demographics with age.

Group	Total samples	Gender distribution	Age range
Colorectal cancer	50	25 females, 25 males	45-75 years
Breast cancer	50	50 females	30-70 years
Prostate cancer	50	50 males	50-80 years
Control group	150	75 females, 75 males	30-80 years

### Blood sample collection and processing

Blood samples (10 mL) were collected in EDTA tubes from each participant. All samples were collected prior to any oncologic treatment (surgery, chemotherapy, or radiotherapy). The samples were centrifuged at 2,000 g for 10 minutes to separate plasma, which was then aliquoted and stored at −80°C until further analysis. All procedures were conducted under sterile conditions to preserve the integrity of the samples.

### RNA isolation

RNA was extracted from plasma using a commercial plasma RNA isolation kit, and protein was extracted using a protein isolation protocol optimized for plasma samples. The purity and concentration of RNA were measured using a Nanodrop spectrophotometer, ensuring an A260/A280 ratio between 1.8 and 2.0 for high-quality RNA.

### Quantitative real-time polymerase chain reaction (qRT-PCR)

The expression levels of SOX2, PIWI proteins, and MALAT1 were assessed using qRT-PCR. Complementary DNA (cDNA) was synthesized from 500 ng of total RNA using a reverse transcription kit (PrimeScript^TM^ RT reagent Kit, for research use only). qRT-PCR was conducted using SYBR Green Master Mix on a QuantStudio 3 Real-Time PCR System. Specific primers [27–30] for SOX2, PIWIL1, PIWIL2, and MALAT1, along with GAPDH as the reference gene [31], were used (Table 2). Relative expression levels were calculated using the 2^(-ΔΔCt) method [32]. qRT-PCR limit of detection (LOD): 0.1 pg/ml, sensitivity 95%.

**Table 2:** RT-PCR primers for SOX2, PIWIL1, PIWIL2, MALAT1 and GADPH.

Gene	Primer Sequence	
SOX2	Forward	CAACATGATGGAGACGGAGC
	Reverse	CTCCGACAAAAGTTTCCACTC
PIWIL1	Forward	ATGGCGTACAGACACGAGGC
	Reverse	AGTGACAGATTTGGCTCTCTG
PIWIL2	Forward	CGAGGCTTGTCTGCTAATCTGG
	Reverse	CTTGTCCACTTCTCTGCTGATACC
MALAT1	Forward	AAAGCAAGGTCTCCCCACAAG
	Reverse	GGTCTGTGCAGATCAAAAGGCA
GADPH	Forward	GCCACATCGCTCAGACAC
	Reverse	TTCACACCCATGACGAACAT

RNA integrity was ensured by including only samples with optimal A260/A280 absorbance ratios (1.8–2.0), consistent with high-purity RNA. To monitor technical consistency, a synthetic RNA spike-in control (miRNeasy QC Spike-in, Qiagen) was added during RNA isolation. Spike-in Ct values remained within ±0.5 cycles across all samples. All qRT-PCR reactions were performed in technical duplicates, with replicate Ct values required to be within a 1-cycle range. Outliers were excluded and reprocessed. Each PCR run included no-template controls and RT-minus controls to exclude contamination and genomic DNA amplification.

### Enzyme-linked immunosorbent assay (ELISA)

To quantify the expression of SOX2 and PIWI proteins at the protein level in plasma, commercial ELISA kits (Fine Biotech Co., Ltd) specific for each target protein were used. Plasma samples were thawed and processed according to the manufacturer’s instructions. The absorbance was measured at 450 nm using a microplate reader “HumaReader HS”, and concentrations were calculated based on standard curves. All samples were run in duplicate to ensure accuracy. ELISA detection range: 0.5 ng/ml – 25 ng/ml, ensuring accurate quantification.

### Immunohistochemistry (IHC) data collection

For each cancer case, data on common IHC diagnostics (Table 3) were collected from clinical records. These included markers routinely used for cancer characterization: Ki-67 (proliferation marker), p53 (tumor suppressor marker), E-cadherin and vimentin (EMT markers), and hormone receptors (estrogen receptor (ER)/progesterone receptor (PR) for breast cancer and androgen receptor (AR) for prostate cancer). The IHC data were used to evaluate the correlation between the blood-based expression levels of SOX2, PIWI proteins, and MALAT1 and the tumor characteristics observed histologically. To ensure consistency and reproducibility in the interpretation of immunohistochemical (IHC) findings, predefined thresholds were applied to classify each marker as positive or negative based on established literature and clinical guidelines. The specific criteria used for each marker in this study are as follows:

**Table 3:** Immunohistochemistry (IHC) profiles in colorectal, breast, and prostate cancer cases.

Marker	Cancer type	Role/Significance	Positive cases (%)	Scoring criteria
Ki-67	Colorectal	Proliferation marker; indicates tumor growth and aggressiveness	84% (42/50)	% of nuclear staining (low/ moderate/high)
	Breast		90% (45/50)	
	Prostate		80% (40/50)	
p53	Colorectal	Tumor suppressor; loss of function suggests poor control	70% (35/50)	Intensity and distribution of nuclear staining
	Breast		60% (30/50)	
	Prostate		--	
E-cadherin	Colorectal	Epithelial marker; loss suggests epithelial-mesenchymal transition (EMT)	76% (38/50)	Completeness and intensity of membrane staining
	Breast		64% (32/50)	
	Prostate		--	
Vimentin	Colorectal	Mesenchymal marker; increase suggests EMT	60% (30/50)	Cytoplasmic staining intensity
	Breast		70% (35/50)	
	Prostate		76% (38/50)	
ER/PR	Colorectal	Hormone receptors; guide treatment decisions and prognosis	--	Positive/negative staining and intensity
	Breast		64% (32/50)	
	Prostate		--	
HER2	Colorectal	Growth factor receptor; amplification indicates poor prognosis	--	Scored 0–3 + : 3+ = positive
	Breast		40% (20/50)	
	Prostate		--	
Androgen receptor	Colorectal	Key androgen-responsive receptor in prostate cancer	--	% and intensity of nuclear staining
	Breast		--	
	Prostate		90% (45/50)

- **Ki-67**: Tumors were considered positive for high proliferative activity if ≥20% of tumor cell nuclei demonstrated positive staining. This threshold aligns with consensus recommendations for high-grade tumors and is commonly applied in breast and colorectal cancer contexts.
- **p53**: Positive expression was defined as >10% of tumor cells exhibiting strong nuclear staining of moderate to high intensity. It is important to note that this threshold may indicate either overexpression of mutant p53 or stabilization of the wild-type protein, and does not by itself imply functional loss of tumor suppressor activity.
- **E-cadherin**: Membranous staining in >70% of tumor cells was considered preserved (positive). Reduced or absent staining below this threshold was interpreted as indicative of epithelial-to-mesenchymal transition (EMT).
- **Vimentin**: A cutoff of >10% cytoplasmic staining in tumor cells was used to define positive expression, in line with EMT-related mesenchymal transformation patterns.
- **Estrogen Receptor (ER)** and **Progesterone Receptor (PR)**: Both markers were considered positive when ≥1% of tumor cell nuclei stained, consistent with the ASCO/CAP guidelines for hormone receptor evaluation in breast cancer.
- **HER2**: Positivity was defined as 3 + membranous staining in ≥10% of tumor cells, based on the 2018 ASCO/CAP scoring criteria for HER2 assessment in breast cancer.
- **Androgen Receptor (AR)**: Nuclear staining in ≥10% of tumor cells was considered positive. This threshold is widely used in both prostate and triple-negative breast cancer research.

All IHC evaluations were conducted by board-certified pathologists using standardized, guideline-based scoring procedures with validated antibodies. Only cases with complete and quality-controlled IHC reports were included, and all assessments were performed independently of the plasma biomarker analysis.

### Statistical analysis

Statistical analysis was performed using SPSS software (version 25.0). Continuous variables were presented as mean ± standard deviation (SD), and categorical variables as frequencies and percentages. Comparison of biomarker levels between cancer and control groups were performed using independent-samples *t*-tests. To control for confounding variables, age and sex were included as covariates in all multivariable logistic regression models assessing the association between biomarker expression (SOX2, PIWI proteins, and MALAT1) and cancer status. Predicted probabilities from these models were then used to construct Receiver Operating Characteristic (ROC) curves, from which sensitivity, specificity, and area under the curve (AUC) values were derived. Association between continuous biomarker expression levels and IHC scores were assessed using Pearson’s correlation coefficients, with reported *p*-values. Statistical significance was set at *p* < 0.05 for all analysis. For all logistic regression models, age and sex were included as covariates, even in cancer subgroups with balanced sex distribution (e.g., colorectal cancer: 25 females and 25 males). This was done to control for potential biological variation in plasma biomarker expression by sex, as well as to ensure accurate estimation of cancer risk associated with each biomarker. Sex was treated as a binary variable (0 = female, 1 = male).

### Sample size justification

A power calculation was conducted using G*Power (version 3.19.7) to assess the adequacy of the sample size. Assuming a medium effect size (Cohen’s *d* = 0.5), a two-tailed alpha of 0.05, and a power of 0.90, the required sample size per group was 44 participants. Our study included 50 participants per cancer group and 150 healthy controls, thus exceeding the minimum required to detect medium-sized effects with adequate power. Additionally, the inclusion of age and sex as covariates in multivariable logistic regression improves model accuracy and further adjusts for potential confounding effects known to influence biomarker levels.

## Results

The study investigated the expression levels of SOX2, PIWI proteins, and MALAT1 in plasma samples from patients with colorectal, breast, and prostate cancers, as well as a control group of healthy individuals. The results were validated at both RNA and protein levels using qRT-PCR and ELISA, respectively, and were further correlated with immunohistochemistry (IHC) data for critical diagnostic markers.

### Plasma expression of SOX2, PIWI proteins, and MALAT1

Significant upregulation of SOX2, PIWI proteins, and MALAT1 mRNA expression levels was observed via qRT-PCR across colorectal, breast and prostate cancers (Table 4, relative expression; *p* < 0.001). **Colorectal cancer**: SOX2 expression was elevated in 76% of colorectal cancer cases (*p* < 0.001), with the highest levels observed in patients with advanced tumor stages. PIWI protein was notably upregulated in 60% of cases (*p* < 0.01), while MALAT1 showed the most pronounced overexpression, with 80% of cases exhibiting significantly higher levels (*p* < 0.001). **Breast cancer**: SOX2 expression was elevated in 60% of breast cancer cases (*p* < 0.001), particularly in HER2-positive subtypes. PIWI expression levels were elevated in 56% of cases (*p* < 0.01), correlating with poor differentiation and proliferation. MALAT1 overexpression was observed in 70% of breast cancer patients (*p* < 0.001), with its highest levels in HER2-positive tumors. **Prostate cancer**: SOX2 expression was upregulated in 72% of prostate cancer cases (*p* < 0.001), particularly in high Gleason score tumors and advanced disease stages. PIWI expression was elevated in 60% of cases (*p* < 0.01), while MALAT1 overexpression was noted in 76% of cases (*p* < 0.001), correlating strongly with androgen receptor (AR) positivity (Table 4). Relative expression values derived from qRT-PCR (2^(-ΔΔCt) method) are dimensionless and reflect fold-changes normalized to GADPH. These results reflect transcript-level differences, not protein concentrations.

**Table 4:** Relative plasma expression of SOX2, PIWI proteins, and MALAT1 by qRT-PCR in cancer and control groups.

Biomarker	Colorectal cancer (Mean Fold Change, 95% CI)	Breast cancer (Mean Fold Change, 95% CI)	Prostate cancer (Mean Fold Change, 95% CI)	Control group (Mean Fold Change, 95% CI)	*p*-value
**SOX2**	4.5 (4.1–4.9)	3.9 (3.6–4.2)	4.2 (3.8–4.2)	1.2 (1.0–1.4)	<0.001
**PIWI proteins**	3.6 (3.2–4.0)	3.2 (2.8–3.6)	3.4 (3.0–3.8)	1.1 (0.9–1.3)	<0.01
**MALAT1**	5.2 (4.8–5.6)	4.8 (4.3–5.2)	5.0 (: 4.5–5.4)	1.3 (1.0–1.5)	<0.001

Based on threshold fold-change values (>2.0), elevated expression of SOX2 was observed in 76% of colorectal, 60% of breast, and 72% of prostate cancer cases. PIWI proteins were upregulated in 60% of colorectal and prostate cancers and in 56% of breast cancers. MALAT1 showed the highest rate of overexpression, with 80% of colorectal, 70% of breast, and 76% of prostate cancer patients exceeding the expression threshold. These proportions confirm the consistency of biomarker elevation across tumor types and align with the mean fold-change values presented in Table 4.

The ELISA results for plasma protein levels were consistent with the qRT-PCR findings. SOX2, PIWI proteins concentrations were significantly higher in cancer cases, confirming their elevated expression at the protein level. The ELISA results confirmed the significantly higher plasma concentrations of SOX2, PIWI proteins in cancer patients compared to healthy controls (Table 5), supporting their elevated expression observed in qRT-PCR analysis. Table 4 reflects relative gene expression (fold-change) at the RNA level, while Table 5 reports absolute protein concentrations in ng/ml as measured by ELISA. This consistency strengthens their potential as reliable diagnostic biomarkers for colorectal, breast, and prostate cancers. Note that MALAT1, as a non-coding RNA, was not quantified by ELISA. Results obtained by ELISA method were consistent across duplicates with intra-assay variation <10%.

**Table 5:** Plasma protein concentrations of SOX2 and PIWI proteins (ELISA results). MALAT1 is a non-coding RNA and was not assessed by ELISA.

Biomarker	Cancer type – Mean concentration (ng/ml) ± SD	Control group – Mean concentration (ng/ml) ± SD	p-value
SOX2	Colorectal cancer – 4.5 ± 0.8	1.2 ± 0.4	*p* < 0.001
	Breast cancer – 3.9 ± 0.6		
	Prostate cancer – 4.2 ± 0.7		
PIWI proteins	Colorectal cancer – 3.6 ± 0.5	1.1 ± 0.3	*p* < 0.01
	Breast cancer – 3.2 ± 0.4		
	Prostate cancer – 3.4 ± 0.5		

### Correlation between plasma biomarkers and IHC markers

The expression of SOX2, PIWI proteins, and MALAT1 in plasma demonstrated strong positive correlations with common IHC markers (Table 6). Correlation coefficients were calculated using Person’s *r*, with statistical significance defined as *p* < 0.05. Analysis were adjusted for age and sex to account for known influences on protein expression.

**Table 6:** Correlation between biomarker expression and IHC markers.

IHC marker	Cancer type	Biomarker	Correlation – r	p-value
Ki-67	Colorectal cancer	SOX2, PIWI	r = 0.72	*p* < 0.001
	Breast cancer	SOX2, PIWI	r = 0.65	*p* < 0.001
	Prostate cancer	SOX2, PIWI	r = 0.68	*p* < 0.001
p53	Colorectal cancer	SOX2	r = 0.60	*p* < 0.01
	Breast cancer	SOX2	r = 0.55	*p* < 0.01
E-cadherin	All cancer types	MALAT1	Negative correlation (↓ E-cadherin)	*p* < 0.001
Vimentin	All cancer types	MALAT1	Positive Correlation (↑ Vimentin)	*p* < 0.001

### Ki-67 (proliferation marker)

SOX2 and PIWI expression in plasma showed a strong correlation with Ki-67 staining, a marker of tumor cell proliferation. This relationship was observed consistently across all cancer types, with correlation coefficients of *r* = 0.72 (*p* < 0.001) in colorectal cancer, *r* = 0.65 (*p* < 0.001) in breast cancer, and *r* = 0.68 (*p* < 0.001) in prostate cancer.

### p53 (tumor suppressor)

SOX2 levels were positively correlated with p53 positivity in colorectal (r = 0.60, p < 0.01) and breast cancer (r = 0.55, p < 0.01), although the functional implications of this association remain uncertain, as p53 IHC cannot distinguish between mutant and wild-type forms. Functional significance should not be inferred without mutation data.

### E-cadherin and vimentin (EMT markers)

MALAT1 overexpression was closely linked to epithelial-mesenchymal transition (EMT), as evidenced by decreased E-cadherin and increased vimentin expression in colorectal, breast, and prostate cancers. This association underscores the role of MALAT1 in driving tumor invasiveness and metastatic spread.

### Hormone receptors

In breast cancer, SOX2 and MALAT1 expression were significantly elevated in ER/PR-negative and human epidermal growth factor receptor 2 (HER2)-positive cases, cancers that are associated with poor prognosis and high metastatic potential. In prostate cancer, MALAT1 expression showed a strong association with AR positivity (90%), particularly in advanced and castration-resistant tumors. Our findings indicate that SOX2 expression in plasma strongly correlates with Ki-67 levels (*r* = 0.72, *p* < 0.001), supporting its role as a marker of tumor cell proliferation. In addition, SOX2 showed a significant positive correlation with p53 expression (*r* = 0.60, *p* < 0.01), suggesting a potential association with tumor suppressor pathway activity, although the functional interpretation of p53 positivity requires caution. Furthermore, MALAT expression was inversely correlated with E-cadherin and positively correlated with vimentin expression (*p* < 0.001), indicating its involvement in epithelial-mesenchymal transition (EMT) and highlighting its potential link to metastatic progression.

### ROC results

To evaluate diagnostic accuracy while controlling for potential confounding by age and sex, ROC analyses were conducted based on predicted probabilities from multivariable logistic regression models. The AUC was highest for SOX2 in colorectal cancer (AUC = 0.89, sensitivity = 84%, specificity = 87%). MALAT1 showed consistent AUC values across all cancers (0.82–0.88), and PIWI proteins ranged from 0.81–0.85. Combined biomarker models yielded AUC > 0.91 in some groups (Table 7). In addition to ROC-derived diagnostic metrics, multivariable logistic regression analysis revealed statistically significant odds ratios for each biomarker, indicating that higher expression levels were strongly associated with cancer status after adjusting for age and sex (Table 8).

**Table 7:** ROC curve results for single and combined biomarker models (adjusted for age and sex). Combined models are based on multivariable logistic regression using biomarker expression levels adjusted for age and sex. In the colorectal cancer group, although the sex distribution was balanced (25 females, 25 males), adjustment for sex was retained to account for possible sex-specific differences in biomarker biology and to improve model precision when comparing against a sex-matched control group. AUC = Area Under the Curve; CI = Confidence Interval.

Model	Cancer Type	AUC (95% CI)	Sensitivity (%)	Specificity (%)	Youden’s Index
SOX2 only	Colorectal	0.89 (0.82-0.95)	84	87	0.71
	Breast	0.83 (0.76-0.90)	78	80	0.58
	Prostate	0.86 (0.80-0.92)	82	85	0.67
PIWI proteins only	Colorectal	0.84 (0.76-0.91)	76	79	0.55
	Breast	0.81 (0.74-0.89)	72	75	0.47
	Prostate	0.85 (0.78-0.91)	78	80	0.58
MALAT1 only	Colorectal	0.88 (0.81-0.93)	82	85	0.67
	Breast	0.82 (0.75-0.89)	78	79	0.57
	Prostate	0.86 (0.79-0.91)	80	84	0.64
SOX2 + MALAT1	Colorectal	0.92 (0.86-0.96)	88	89	0.77
	Breast	0.87 (0.81-0.92)	83	85	0.68
	Prostate	0.90 (0.85-0.95)	85	88	0.73
SOX2 + PIWI	Colorectal	0.91 (0.85-0.95)	85	87	0.72
	Breast	0.86 (0.80-0.91)	80	83	0.63
	Prostate	0.88 (0.83-0.93)	83	85	0.68
PIWI + MALAT1	Colorectal	0.90 (0.84-0.94)	84	85	0.69
	Breast	0.85 (0.79-0.90)	80	81	0.61
	Prostate	0.89 (0.83-0.94)	84	86	0.71
SOX2 + PIWI + MALAT1	Colorectal	0.94 (0.89-0.97)	90	91	0.81
	Breast	0.89 (0.84-0.93)	85	86	0.71
	Prostate	0.92 (0.87-0.95)	87	89	0.76

**Table 8:** Odds ratios (Ors) for cancer diagnosis per unit increase in biomarker expression (adjusted for age and sex).

Biomarker	Cancer Type	OR (95% CI)	*p*-value
SOX2	Colorectal	4.20 (2.40-7.36)	<0.001
	Breast	3.65 (2.02-6.58)	<0.001
	Prostate	4.01 (2.25-7.15)	<0.001
PIWI	Colorectal	3.10 (1.75-5.52)	<0.001
	Breast	2.85 (1.50-5.42)	<0.001
	Prostate	3.28 (1.80-5.97)	<0.001
MALAT1	Colorectal	4.75 (2.60-8.70)	<0.001
	Breast	3.95 (2.18-7.10)	<0.001
	Prostate	4.20 (2.40-7.36)	<0.001

## Discussion

This study evaluated the expression levels of SOX2, PIWI proteins, and MALAT1 in plasma samples from colorectal, breast, and prostate cancer patients, as well as healthy controls. The findings, validated at both RNA and protein levels, demonstrated significant biomarker upregulation in cancer cases, with strong correlations to key IHC markers. These results highlight the potential of SOX2, PIWI proteins, and MALAT1 as diagnostic and prognostic biomarkers for aggressive cancers. These associations remained significant after adjusting for age and sex, supporting their robustness across demographic variables. To ensure that these associations were not confounded by demographic variability, all statistical models were adjusted for age and sex, both of which are known to influence circulating biomarker levels. Although tissue-based comparisons were not performed in this study, we implemented rigorous plasma RNA quality control measures – including spectrophotometric purity assessment, synthetic spike-in RNA controls, and technical duplicate reproducibility – to ensure the reliability and reproducibility of expression data derived from plasma samples.

### Elevated expression of SOX2, PIWI proteins, and MALAT1

The study revealed a significant increase in the expression of SOX2, PIWI proteins, and MALAT1 in all three cancer types when compared to controls:

- **SOX2**: Elevated expression in **76%** of colorectal, **60%** of breast, and **72%** of prostate cancers. SOX2 levels correlated strongly with advanced tumor stages [33]. Its role in maintaining stem cell pluripotency and promoting cancer cell proliferation aligns with previous findings [10,21] associating SOX2 with cancer aggressiveness and therapy resistance. In multivariable models, SOX2 demonstrated the highest diagnostic performance in colorectal cancer, with an AUC of 0.89 (95% CI: 0.82–0.95), sensitivity of 84% and specificity of 87%. Although the highest SOX2 expression levels were observed in patients later confirmed to have advanced-stage disease, complete TNM staging data were not uniformly available for all participants. Therefore, we cannot currently establish a robust stage-specific expression trend and caution against overinterpretation in this regard.
- **PIWI proteins**: PIWI proteins were notably upregulated across cancers (60% in colorectal and prostate, 56% in breast cancer). Their correlation with Ki-67 staining indicates a role in enhancing tumor proliferation, as PIWI proteins are known to support stem cell self-renewal and genomic stability [34–36].
- **MALAT1**: Among the three biomarkers, MALAT1 showed the highest overexpression, particularly in colorectal cancer (80%, *p* < 0.001). Its upregulation across cancers was closely linked to epithelial-mesenchymal transition (EMT), as evidenced by decreased E-cadherin and increased vimentin levels. This reinforces MALAT1’s role in promoting invasiveness and metastatic spread [37]. MALAT1 also demonstrated consistent diagnostic performance across cancers, with AUCs between 0.82–0.88. Previous studies have also identified MALAT1 as a promising plasma-based biomarker beyond the tumor types evaluated here. A sensitivity of 88.89% and specificity of 66.67% for MALAT1 in diagnosing epithelial malignant pleural mesothelioma were reported [17], highlighting its potential cross-cancer diagnostic relevance.

The consistency between qRT-PCR and ELISA data strengthens the reliability of these findings, confirming elevated biomarker concentrations at both RNA and protein levels. Combined biomarker models (e.g., SOX+MALAT1) further improved diagnostic discrimination, with AUCs exceeding 0.90 in colorectal and prostate cancers.

Given the challenges inherent in using plasma-derived RNA, additional technical validation steps were implemented to ensure the reliability of our results. A known limitation in plasma-based qRT-PCR analysis is RNA degradation and sample-to-sample variability due to pre-analytical factors. To address this, only high-purity RNA samples (A260/A280 ratio 1.8–2.0) were included. A synthetic spike-in RNA control was used to monitor RNA extraction and cDNA synthesis efficiency, and all Ct values remained within a narrow acceptable range across the study. Reactions were run in technical duplicates, and outlier replicates were repeated. These quality control measures strengthen confidence in the robustness and reproducibility of our reported biomarker expression levels.

### Correlation with IHC markers

The significant associations between plasma biomarkers and IHC markers provide further insight into their roles in tumor biology:

- **Ki-67 (Proliferation)**: SOX2 and PIWI expression levels showed a strong positive correlation with Ki-67 staining across all cancer types (*r* = 0.65–0.72, *p* < 0.001), highlighting their association with tumor cell proliferation and aggressive behavior [11,13].
- **p53 (Tumor suppressor)**: SOX2 expression was significantly elevated in p53-positive colorectal (*r* = 0.60, *p* < 0.01) and breast cancers (*r* = 0.55, *p* < 0.01), suggesting a role for SOX2 in tumors with impaired p53 function. This association may reflect the disruption of tumor suppression mechanisms driving disease progression [38]. While SOX2 was positively correlated with p53 IHC positivity in colorectal and breast cancers, this finding should be interpreted with caution. p53 IHC staining reflects protein accumulation but does not differentiate between overexpression of mutant p53 and stabilization of wild-type protein. Thus, conclusions regarding p53 pathway impairment would require confirmatory molecular analyses, such as TP53 mutation status.
- **E-cadherin and vimentin (EMT markers)**: MALAT1 expression demonstrated a negative correlation with E-cadherin and a positive correlation with vimentin (*p* < 0.001), consistent across all cancer types. These findings emphasize MALAT1’s involvement in EMT, a key process facilitating tumor invasiveness and metastasis [37]. Although direct correlation between plasma and matched tumor expression levels of SOX2, PIWI proteins, and MALAT1 was not assessed in this study, prior research supports their concordance in several cancers. Future studies incorporating parallel tissue-plasma profiling are necessary to validate and refine the diagnostic relevance of these circulating biomarkers.
- **Hormone receptors**: In breast cancer, SOX2 and MALAT1 were upregulated in ER/PR-negative and HER2-positive cases, reflecting their association with more aggressive phenotypes [11,37]. In prostate cancer, MALAT1 expression strongly correlated with androgen receptor (AR) positivity (90%, *p* < 0.001) [39].

The identification of SOX2, PIWI proteins, and MALAT1 as plasma-based biomarkers represents a promising direction for the non-invasive detection and molecular characterization of colorectal, breast, and prostate cancers; however, further studies incorporating stage-specific analyses are needed to determine their utility in early detection. Although transcription factors and non-coding RNAs are intracellular by nature, several studies have demonstrated their stable detectability in plasma or serum—primarily due to passive release during tumor cell death or active export via extracellular vesicles. Prior research confirms the presence of SOX2, PIWI proteins, and MALAT1 in plasma of cancer patients, supporting their relevance as non-invasive biomarkers. While our findings demonstrate significant differential expression and strong correlations with clinically relevant markers, the current study was not designed to assess biomarker performance in early-stage or asymptomatic populations are warranted to validate this application. Given their non-invasive nature, these biomarkers offer a promising alternative to conventional diagnostic methods, which often rely on invasive biopsies and imaging techniques. Their ability to correlate with key tumor markers, such as Ki-67 and p53, suggest a potential role on screening high-risk populations, enabling earlier diagnosis and more effective patient stratification for timely interventions. However, large-scale validation studies are necessary to confirm these findings and to define precise clinical cutoff values. By complementing existing screening programs, plasma biomarker testing could bridge diagnostic gaps, particularly in case where traditional histopathological assessments are impractical or inconclusive. Their use in routine clinical practice may help improve early detection rates, leading to earlier treatment initiation and better patient outcomes. Beyond their diagnostic utility, the adoption of plasma-based biomarkers holds significant economic benefits. By reducing dependence on costly and resource-intensive procedures, such as magnetic resonance imaging scans and invasive biopsies, this approach could lower healthcare expenditures and make cancer screening more accessible, especially in resource-limited settings. This is particularly relevant for countries with constrained oncology infrastructure, where timely and affordable diagnosis remains a major challenge.

## Conclusion

The differential expression of SOX2, PIWI proteins, and MALAT1 between cancer patients and healthy controls supports their potential utility as plasma-based biomarkers for distinguishing cancer cases from non-cancer cases. These findings support the potential of SOX2, PIWI proteins, and MALAT1 as non-invasive plasma biomarkers in cancer patients. However, future studies with larger, stage-stratified cohorts are essential to validate their clinical performance for early diagnosis, prognosis, and treatment monitoring.

### Limitations

Despite the promising findings of our study, several limitations must be addressed before these biomarkers can be fully integrated into clinical practice. This study was conducted within a single-center setting, introducing a potential selection bias that may limit the generalizability of results. Therefore, multi-center validation studies are essential to confirm the robustness of these findings across diverse populations and clinical settings. Additionally, the lack of longitudinal data presents another challenge, as the current study does not assess biomarker stability over time. Future research should focus on longitudinal follow-up studies to determine whether these biomarkers maintain their predictive value across different disease stages and treatment responses. Finally, while these biomarkers show strong correlations with tumor markers, further functional studies are required to elucidate their precise mechanistic roles in tumorigenesis and metastasis. Investigating the molecular pathways involving SOX2, PIWI proteins, and MALAT1 could provide deeper insights into their role in cancer progression, potentially paving the way for targeted therapeutic strategies. Cancer stage data were not uniformly available for all enrolled cases, limiting the ability to evaluate biomarker performance across disease progression. Stage-specific analyses will be critical in future studies aiming to determine utility in early detection and clinical stratification. In conclusion, the findings of this study underscore the potential of plasma-based biomarkers in cancer diagnostics, but further research is needed to validate their clinical applicability. Through multi-center validation, longitudinal follow-ups, and mechanistic studies, these biomarkers could eventually be incorporated into routine cancer screening and management, facilitating the development of early detection and improving patient outcomes.

### Declaration of generative AI and AI-assisted technologies

During the preparation of this paper the authors used ChatGPT (*https://chat.openai.com/*) for stylistic purpose, checking grammar and spelling. After using this tool the authors reviewed and edited the content, they take full responsibility for the content of the publication.
